# Global burden of trichomoniasis: current status, trends, and projections (1990–2021)

**DOI:** 10.3389/fpubh.2025.1530227

**Published:** 2025-02-28

**Authors:** Xingmin Wei, Lu Liu, Kun Liu, Xiaoang Qin, Jianjun Wu, Lou Jiang, Longjian Shang

**Affiliations:** ^1^School of Public Health, Gansu of Chinese Medicine, Lanzhou, China; ^2^The Collaborative Innovation Center for Prevention and Control by Chinese Medicine on Disease Related Northwestern Environment and Nutrition, Lanzhou, China; ^3^Department of Epidemiology, School of Public Health, Air Force Medical University, Xi’an, China

**Keywords:** trichomoniasis, global burden, trend analysis, sexually transmitted disease, annual percentage change

## Abstract

**Objectives:**

Trichomoniasis one of the most neglected sexually transmitted diseases (STDs), imposes a significant global disease burden. This study aims to assess the burden and trends of trichomoniasis from 1990 to 2021 and to project its incidence from 2022 to 2050.

**Methods:**

This study utilized data from the Global Burden of Disease 2021 study for secondary analysis. We determined the age-standardized incidence rate (ASIR) and disability-adjusted life years (DALYs) of trichomoniasis by sex, age, and socio-demographic index (SDI) level. Changes in burden trends across sex and age were explored from 1990 to 2021, using joinpoint regression. The incidence of trichomoniasis was projected for the period 2022 to 2050, using R software.

**Results:**

From 1990 to 2021, the estimated annual percentage change (EAPC) in the global ASIR of trichomoniasis was 0.09 (95% CI: 0.06 to 0.13). In 2021, the global ASIR of trichomoniasis was 4,133.41 per 100,000 people (95% UI: 3,111.37 to 5,583.56 per 100,000). By population group, the ASIR was higher in men (4,353.43 per 100,000) than in women (3,921.31 per 100,000) in 2021, while the DALY rate was significantly higher in women than in men (6.45 vs. 0.23 per 100,000). When divided by age groups, the trend in ASIR among women aged 30–54 years aligned closely with the overall population incidence trend. In 2021, ASIRs were highest in low SDI regions, and the projected ASIRs by 2050 are 5,680.57 per 100,000 in males and 5,749.47 per 100,000 in females.

**Conclusion:**

Trichomoniasis represents a significant global disease burden, particularly among women in low-income areas and individuals aged 30–54 years. The study highlights the need for targeted strategies to reduce the burden of trichomoniasis-related infections, especially in vulnerable populations.

## Introduction

Trichomoniasis is a sexually transmitted disease (STD) caused by *Trichomonas vaginalis* (1), which spreads primarily through sexual contact, although rare cases involve transmission *via* contaminated objects (2). Traditionally, a diagnosis is confirmed by detecting Trichomonas in vaginal discharge under a microscope. However, in recent years, it has also been possible to obtain a diagnosis through a single rapid nucleic acid diagnostic test (3). Among symptomatic patients, the most common clinical manifestations are female vaginitis and male urethritis (4), though most men with Trichomonas infection are asymptomatic (5). Additionally, Trichomonas infection increases the risk of human immunodeficiency virus (HIV) transmission, other sexually transmitted infections, pelvic inflammatory disease, and cervical cancer (6, 7). Although trichomoniasis is a relatively common sexually transmitted disease with harmful effects, there is currently no regular screening for any population group except women living with HIV.

According to the latest estimates of the World Health Organization, in 2020, the incidence rate of trichomoniasis among those aged 15–49 was 38.0 per 1,000 in females and 41.0 per 1,000 in males (8). There were 156 million new cases of *Trichomonas vaginalis* infection (73.7 million in females and 82.6 million in males) (9). A study in Nigeria revealed that the highest prevalence of trichomoniasis (31.9%) was found among individuals aged 38–47 years (39.3%), married adults (76.3%), and those with secondary education (68.9%) (10). A study conducted at a Spanish tertiary care hospital reported a *Trichomonas vaginalis* prevalence of 0.8%, though fewer than 20% of patients with trichomoniasis were fully screened for other genital pathogens (11). These findings highlight the growing burden of trichomoniasis and underscore the importance of integrating routine vaginal testing for trichomoniasis with gynecological examinations.

Although previous studies have provided valuable insights into the prevalence of trichomoniasis, they have not comprehensively examined long-term trends in its global burden. While the disease is increasingly recognized as a significant yet underemphasized STD, there remains a gap in understanding its temporal dynamics across different regions and populations. Furthermore, while existing data highlight the high burden of trichomoniasis, especially in certain demographic groups, there is limited research on projecting future trends in its incidence.

To address the gaps in understanding the long-term trends and future projections of trichomoniasis, we conducted a comprehensive analysis based on the 2021 Global Burden of Disease (GBD) study.^1^ We extracted data on trichomoniasis incidence and population from this database for the years 1990–2021, with two primary objectives: first, to explore trends in the global and regional burden of trichomoniasis from 1990 to 2021; and second, to project the burden of trichomoniasis incidence through 2050. By analyzing these trends, we aim to provide valuable insights into how the burden of trichomoniasis may evolve in the coming decades. Our findings will help guide public health efforts, inform policy decisions, and shape targeted interventions necessary to address the rising global burden of trichomoniasis, especially in vulnerable populations.

## Methods

### Data source

The burden of disease data used in this study were obtained from the Global Burden of Disease (GBD) 2021 study (see text footnote 1). The dataset is initiated and coordinated by the Institute for Health Metrics and Evaluation (IHME). It is a highly open and important resource that can be freely accessed and used by researchers worldwide, strongly promoting international exchanges and development in health research. The dataset has extensive data sources, covering national health statistics, reports from medical institutions, epidemiological surveys, census data, etc. The GBD 2021 has outstanding achievements. It has evaluated 369 diseases and 87 risk factors in 204 countries and 21 regions (12, 13) and these regions are subdivided into 5 SDI segments according to the Socio-Demographic Index (SDI) (14).

This dataset covers the period from 1990 to 2021 and encompasses numerous health indicators such as incidence, prevalence, mortality, disability-adjusted life years, maternal mortality, and exposure situations. It is of great significance in many fields, including public health policy-making, disease prevention and control strategy planning, health economics research, and epidemiological analysis. It builds a unified and standardized platform for researchers from various countries, greatly promotes global health research, facilitates cross-national and cross-regional comparisons and collaborations, and elevates the international exchanges in global health research to a new level.

In this study, the indicators of interest were selected from the GBD database, including data on the number of trichomoniasis cases, incidence rate, Disability-Adjusted Life Years (DALYs), and DALY rates between 1990 and 2021. These data were classified by gender, age, SDI groups, and regions. The age distribution was divided into <20 years old, >85 years old, and every five year intervals between 20 and 85 years old.

### Evaluation indicators

The age-standardized rate (ASR), is designed to eliminate the influence of different age compositions when comparing incidence rates, DALYs (Disability-Adjusted Life Years), and other data among different populations. Among them, α_i_ and w_i_, respectively, stand for the age-specific rate and weight of the i-th age group (15).

$$ASR=\frac{\Sigma_{i=1}^{N}a_{i}w_{i}}{\Sigma_{i=1}^{N}w_{i}}\times 100000$$

The Socio-Demographic Index (SDI) provides a comprehensive picture of the development of a country or region. It includes indicators such as the average educational level of women under 25 years old and per capita income.

The Estimated Annual Percentage Change (EAPC) is an indicator used to describe the changes in the ASR between 1990 and 2021. It can demonstrate the long-term trends of the ASR in relation to the disease burden of trichomoniasis. Specifically, the EAPC is obtained by fitting the natural logarithm of the ASR to the calendar years and then applying a regression model. In this formula, *β* represents the positive or negative trend of the ASR (15). This study used EAPC with 95% confidence intervals (CI) to represent annual changes in ASR from 1990 to 2021 (16).

$$\mathrm{EAPC}=\left(\exp\left(\beta \right)-1\right)\times 100$$

### Statistical analysis

EAPC was used in this study to quantify trends in the burden of trichomoniasis. Changes in the age-standardized incidence rate (ASIR) were used to inform prevention strategies.

Joinpoint regression analyses were performed with Joinpoint 5.0 software from the American National Cancer Institute.^2^ The software was used to calculate the annual percentage change (APC) to characterize trends in the burden of disease across multiple phases (17).

The Monte Carlo permutation test was applied to evaluate the computational results of the software (18), with the significance level set at *α* = 0.05. If the *p*-value is less than 0.05, it indicates that the difference in results is statistically significant at the 5% significance level.

$$APC_{i}=\left(e^{\beta_{i}}-1\right)\times 100$$

Compared with other methods, APC modeling is more flexible in parameter selection and prior probability distribution, making it more robust and reliable for prediction (19). Therefore, this study used the age-period cohort (APC) model to project the incidence of trichomoniasis by 2050. In the APC model, β_i_ reflects the degree and direction of the impact of certain potential influencing factors in the i-th age group on the studied indicator.

All statistical analyses and data visualizations were performed in R (version 4.3.2), using the BAPC and ggplot2 packages. Map production was done using ArcGIS 10.8 system software. Subgroup analyses were conducted according to gender, age, SDI, and the 21 geographic locations. A *p*-value of <0.05 was considered statistically significant.

## Results

### Estimated global burden of trichomoniasis

The incidence of trichomoniasis has been increasing globally from 1990 to 2021, with a notable rise in 2021. In that year, the global ASIR of trichomoniasis infection was 4,133.41 per 100,000 people (95% UI: 3,111.37/100,000 to 5,583.56/100,000) (Table 1). In 2021, the total number of trichomoniasis cases was 3,419.16×10^5^ (95% UI: 2,580.46×10^5^ to 4,642.95×10^5^), compared to 1,978.37×10^5^ cases in 1990, representing a 73% increase from 1990 to 2021. The DALY rates for trichomoniasis showed a stable trend from 1990 to 2021, with a global ASR for DALY of 3.33 per 100,000 (95% UI: 1.37/100,000 to 7.12/100,000) in 2021 (Supplementary Table S1).

**Table 1:** Number of trichomoniasis cases, age-standardized incidence rates, and their temporal trends for 1990 and 2021.

Characteristics	1990		2021		1990 to 2021
	No. of cases ×10^5^ (95% UI)	ASIR/100,000 (95% UI)	No. of cases ×10^5^ (95% UI)	ASIR/100,000 (95% UI)	EAPC (95% CI)
Global	1,978.37 (1,473.58 to 2,635.38)	3,950.01 (2,966.44 to 5,317.01)	3,419.16 (2,580.46 to 4,642.95)	4,133.41 (3,111.37 to 5,583.56)	0.09 (0.06 to 0.13)
Sex					
Female	912.83 (671.57 to 1,219.17)	3,635.06 (2,678.42 to 4,937.63)	1,604.35 (1,195.69 to 2,194.96)	3,921.31 (2,904.04 to 5,303.8)	0.15 (0.07 to 0.23)
Male	1,065.54 (788.85 to 1403.34)	4,265.25 (3,187.26 to 5,667.53)	1,814.81 (1,360.01 to 2,433.99)	4,353.43 (3,251.82 to 5,818.73)	0.05 (0.04 to 0.06)
Age					
<20 years	43.57 (23.92 to 73.12)	192.92 (105.92 to 323.74)	60.08 (33.21 to 100.54)	227.94 (125.99 to 381.44)	0.50 (0.36 to 0.64)
20–24 years	155.92 (85.30 to 246.76)	3,168.53 (1,733.35 to 5,014.6)	206.29 (113.56 to 324.92)	3,454.52 (1,901.61 to 5,441.03)	0.16 (0.10 to 0.22)
25–29 years	286.51 (159.68 to 459.28)	6,472.95 (3,607.65 to 10,376.41)	401.28 (224.09 to 639.68)	6,820.47 (3,808.76 to 10,872.64)	0.08 (0.03 to 0.13)
30–34 years	359.71 (192.65 to 558.22)	9,332.9 (4,998.43 to 14,483.31)	583.31 (312.22 to 905.40)	9,649.85 (5,165.09 to 14,978.31)	0.06 (0.01 to 0.10)
35–39 years	375.33 (215.35 to 577.47)	10,655.47 (6,113.51 to 16,393.93)	620.05 (353.63 to 950.10)	11,055.31 (6,305.12 to 16,939.89)	0.08 (0.03 to 0.12)
40–44 years	288.51 (162.65 to 484.88)	10,070.71 (5,677.44 to 16,925.28)	529.15 (295.72 to 893.85)	10,577.79 (5,911.49 to 17,867.94)	0.11 (0.07 to 0.15)
45–49 years	190.74 (110.27 to 347.59)	8,214.65 (4,749.04 to 14,969.64)	404.88 (234.29 to 742.67)	8,550.71 (4,947.9 to 15,684.44)	0.10 (0.07 to 0.13)
50–54 years	125.43 (75.22 to 201.24)	5,900.63 (3,538.37 to 9,467.13)	273.77 (164.10 to 438.94)	6,153.31 (3,688.36 to 9,865.6)	0.10 (0.07 to 0.12)
55–59 years	68.04 (43.24 to 102.30)	3,673.8 (2,334.97 to 5,523.67)	150.14 (95.59 to 225.60)	3,794.13 (2,415.52 to 5,700.99)	0.08 (0.07 to 0.10)
60–64 years	35.51 (26.10 to 45.97)	2,210.66 (1,625.21 to 2,862.41)	72.82 (52.75 to 93.98)	2,275.22 (1,647.91 to 2,936.30)	0.07 (0.06 to 0.08)
65–69 years	23.41 (17.18 to 30.30)	1,894 (1,390.24 to 2,451.6)	53.99 (39.03 to 69.54)	1,957.14 (1,414.92 to 2,520.96)	0.08 (0.07 to 0.09)
70–74 years	13.53 (9.91 to 17.51)	1,598.2 (1,170.4 to 2,068.69)	33.43 (24.17 to 43.11)	1,624.19 (1,174.16 to 2,094.59)	0.08 (0.06 to 0.09)
75–79 years	7.67 (5.61 to 9.94)	1,246.61 (912.1 to 1,615.59)	17.38 (12.57 to 22.45)	1,318.11 (953.1 to 1,702.32)	0.10 (0.08 to 0.12)
80–84 years	3.32 (2.42 to 4.30)	938.9 (684.05 to 1,214.61)	8.66 (6.25 to 11.20)	988.6 (713.96 to 1,278.94)	0.14 (0.11 to 0.18)
85+ years	1.17 (0.85 to 1.51)	572.94 (416.4 to 740.44)	3.91 (2.81 to 5.07)	566.73 (406.95 to 733.63)	−0.01 (−0.02 to 0.00)
SDI					
Low SDI	235.83 (178.23 to 309.84)	6,292.81 (4,797.72 to 8,330.93)	582.18 (435.76 to 766.43)	6,376.84 (4,831.97 to 8,522.19)	−0.01 (−0.05 to 0.03)
Low-middle SDI	355.16 (264.95 to 475.11)	3,731.32 (2,809.68 to 5,013.14)	748.79 (559.32 to 1005.17)	3,905.3 (2,943.09 to 5,265.6)	0.06 (0.03 to 0.1)
Middle SDI	682.27 (506.23 to 906.80)	4,318.11 (3,246.91 to 5,852.54)	1,146.44 (863.87 to 1575.99)	4,210.06 (3,165.35 to 5,731.87)	−0.14 (−0.17 to −0.1)
High-middle SDI	377.53 (280.16 to 507.65)	3,408.26 (2,557.88 to 4,605.36)	525.25 (397.90 to 728.16)	3,432.79 (2,574.18 to 4,650.02)	−0.04 (−0.08 to 0.00)
High SDI	325.55 (242.53 to 439.54)	3,329.82 (2,489.07 to 4,499.36)	413.49 (314.08 to 565.84)	3,404.25 (2,546.16 to 4,635.24)	0.07 (0.03 to 0.11)
Region					
Andean Latin America	13.23 (9.83 to 17.40)	4,216.13 (3,179.11 to 5,573.02)	28.86 (21.61 to 38.26)	4,196.55 (3,165.43 to 5,586.06)	−0.02 (−0.04 to 0)
Australasia	6.14 (4.55 to 8.12)	2,773.89 (2,062.72 to 3,662.82)	9.45 (7.17 to 12.60)	2,798.91 (2,088.95 to 3,714.53)	0.03 (0 to 0.06)
Caribbean	16.75 (12.49 to 22.15)	5,144.85 (3,875.23 to 6,812.81)	25.85 (19.56 to 34.43)	5,160.81 (3,871.94 to 6,892.84)	0.03 (0.02 to 0.05)
Central Asia	24.12 (17.69 to 31.61)	3,904.12 (2,910.89 to 5,178.65)	39.81 (29.50 to 52.84)	3,917.34 (2,933.07 to 5,188.98)	−0.01 (−0.04 to 0.02)
Central Europe	44.07 (33.06 to 59.29)	3,283.16 (2,452.52 to 4,386.95)	43.46 (33.17 to 59.43)	3,317.85 (2,480.38 to 4,474.58)	0.04 (0.02 to 0.06)
Central Latin America	90.25 (65.98 to 119.70)	6,554 (4,918.3 to 8,860.03)	174.30 (130.73 to 236.55)	6,478.6 (4,864.22 to 8,776.00)	0.02 (−0.01 to 0.06)
Central Sub-Saharan Africa	22.98 (16.93 to 30.46)	5,710.58 (4,288.72 to 7,674.52)	62.33 (46.39 to 83.48)	5,715.45 (4,291.68 to 7,667.09)	0.01 (−0.01 to 0.04)
East Asia	495.11 (365.37 to 665.88)	4,013.34 (3,001.07 to 5,447.09)	673.59 (509.43 to 933.96)	3,884.66 (2,907.45 to 5,288.34)	−0.16 (−0.23 to −0.09)
Eastern Europe	70.57 (52.78 to 95.99)	2,848.13 (2,109.38 to 3,899.4)	68.47 (51.43 to 94.56)	2,834.63 (2,112.48 to 3,873.47)	0.02 (0 to 0.03)
Eastern Sub-Saharan Africa	133.50 (101.74 to 173.81)	9,935.17 (7,591.06 to 13,264.03)	334.77 (253.24 to 439.65)	9,845.31 (7,505.32 to 13,263.53)	−0.06 (−0.11 to −0.01)
High-income Asia Pacific	58.25 (43.84 to 80.22)	2,992.28 (2,251.3 to 4,055.4)	60.78 (46.90 to 82.89)	2,980.3 (2,233.1 to 4,050.67)	−0.02 (−0.04 to 0)
High-income North America	133.40 (98.96 to 180.99)	4,242.67 (3,184.67 to 5,759.77)	163.16 (123.19 to 224.40)	4,178.27 (3,117.91 to 5,751.7)	−0.05 (−0.11 to 0.01)
North Africa and Middle East	97.28 (72.61 to 128.37)	3,652.23 (2,759.8 to 4,848.42)	238.44 (177.46 to 322.09)	3,617.82 (2,727.47 to 4,861.82)	−0.18 (−0.24 to −0.12)
Oceania	4.05 (3.03 to 5.26)	7,251.53 (5,505.37 to 9,380.05)	9.40 (7.03 to 12.33)	7,141.29 (5,407.19 to 9,356.59)	0.17 (0.1 to 0.24)
South Asia	244.94 (181.04 to 329.78)	2,664.45 (2,001.7 to 3,624.9)	505.83 (376.24 to 688.75)	2,640.4 (1,970.03 to 3,591.75)	−0.12 (−0.16 to −0.07)
Southeast Asia	180.01 (132.88 to 240.42)	4,381.04 (3,299.41 to 5,875.38)	329.17 (247.16 to 448.93)	4,287.78 (3,226.56 to 5,799.16)	−0.04 (−0.06 to −0.02)
Southern Latin America	13.45 (9.99 to 17.64)	2,805.90 (2,083.22 to 3,697.66)	21.05 (15.81 to 28.22)	2,842.95 (2,123.02 to 3,806.04)	0.02 (−0.01 to 0.05)
Southern Sub-Saharan Africa	41.02 (30.81 to 53.92)	9,256.16 (6,955.59 to 12,297.03)	72.40 (53.50 to 96.53)	8,595.4 (6,436.05 to 11,517.48)	−0.59 (−0.79 to −0.38)
Tropical Latin America	77.50 (57.52 to 104.36)	5,589.86 (4,192.39 to 7,653.03)	143.08 (106.79 to 198.86)	5,551.14 (4,156.53 to 7,669.63)	−0.02 (−0.04 to 0)
Western Europe	100.58 (75.86 to 133.50)	2,377.91 (1,784.38 to 3,191.56)	112.28 (86.56 to 150.84)	2,379.95 (1,788.17 to 3,183.94)	0.01 (−0.01 to 0.03)
Western Sub-Saharan Africa	111.16 (83.16 to 145.78)	7,683.88 (5,873.39 to 10,177.72)	302.68 (225.21 to 399.73)	7,912.01 (6,019.8 to 10,595.35)	−0.09 (−0.15 to −0.02)

**Figure 1:**
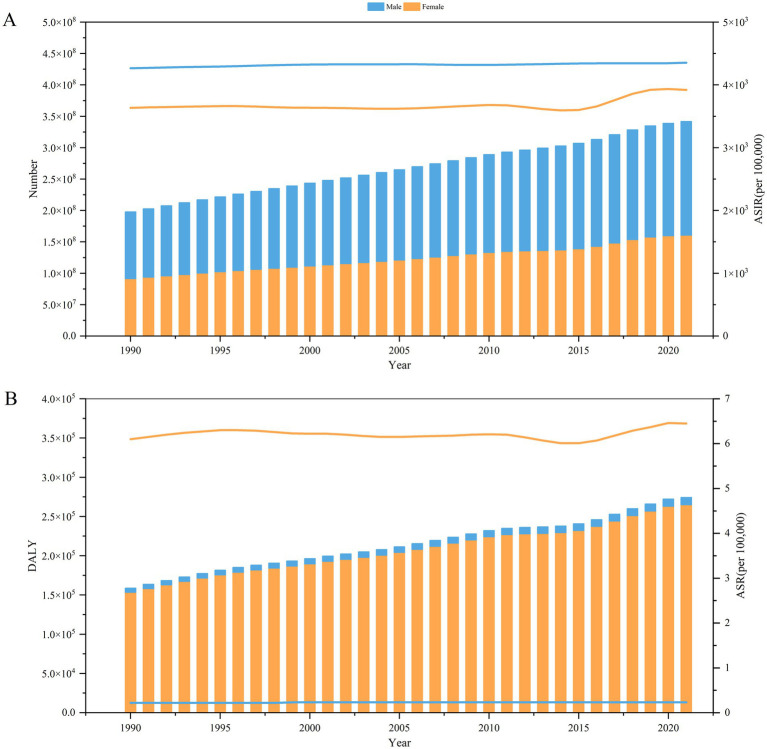
Burden of trichomoniasis by sex, 1990–2021. **(A)** Number of trichomoniasis cases (left) and ASIRs (right) by sex. **(B)** Trichomoniasis DALY (left) and ASR for DALY (right) by sex. ASIR, age-standardized incidence rate; DALY, disability-adjusted life year; ASR, age-standardized rate; SDI, Socio-demographic Index.

From 1990 to 2021, men always had more cases of trichomoniasis infections than women did (Figure 1A), and the number of cases of trichomoniasis infections in men peaked at 1,814.81×10^5^ (95% UI: 1,360.01×10^5^ to 2,433.99×10^5^) in 2021. Meanwhile a similarly higher ASIR in men compared to women, ASIR for men tops out at 4,353.43/100,000 (95% UI: 3,251.82/100,000 to 5,818.73/100,000) in 2021. However, the DALY and ASR for DALY are much higher for females than for males (Figure 1B), and the ASR of DALY for women was highest in 2021 at 6.45/100,000 (95% UI: 2.65/100,000 to 13.87/100,000).

The number of cases of trichomoniasis infections from 1990 to 2021 increased in all age groups, while the EAPC was −0.01 (95% CI: −0.02 to 0.00) in the 85+ age group and positive in the rest of the age groups (Table 1). Indicates that except for the 85+ age group, ASIR for the rest of the population showed an overall increasing trend over the 30 years, with the fastest increase occurring in the <20 years age group. The three highest ASIRs for trichomonas infections in 2021 were 35 to 39 years (11,055.31/100,000), 40–44 years (10,577.79/100,000) and 30–34 years (9,649.85/100,000). DALY for trichomonas infection increased in all age groups from 1990 to 2021 (Supplementary Table S1).

**Figure 2:**
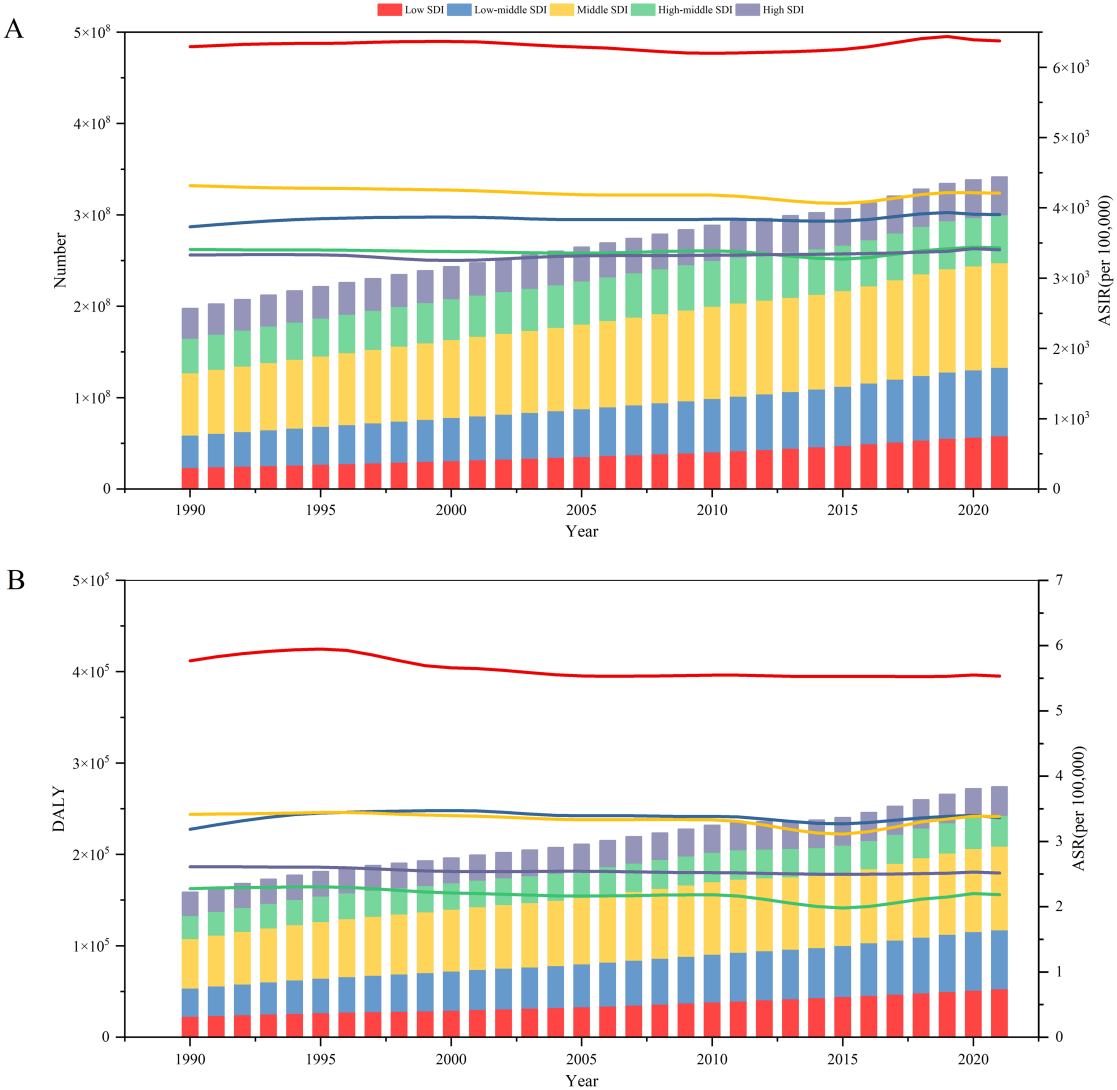
Burden of trichomoniasis by SDI, 1990–2021. **(A)** Number of trichomoniasis cases (left) and ASIRs (right) by SDI subgroup. **(B)** Trichomoniasis DALY (left) and ASR for DALY (right) by SDI subgroup. ASIR, age-standardized incidence rate; DALY, disability-adjusted life year; ASR, age-standardized rate; SDI, Socio-demographic Index.

The incidence of cases increased in all SDI regions over 32 years (Figure 2A). The highest increase in cases was in the low SDI region (147%), and the lowest in the high SDI region (27%). EAPC were positive in low-middle SDI and high SDI trichomoniasis ASIRs, and negative in the remaining SDI regions (Table 1). DALY for trichomoniasis infections from 1990 to 2021 increased in all five SDI regions, but the EAPC was negative in all SDI areas (Supplementary Table S1). This indicates a downward trend in ASR for DALY in all SDI regions over the last 32 years (Figure 2B).

**Figure 3:**
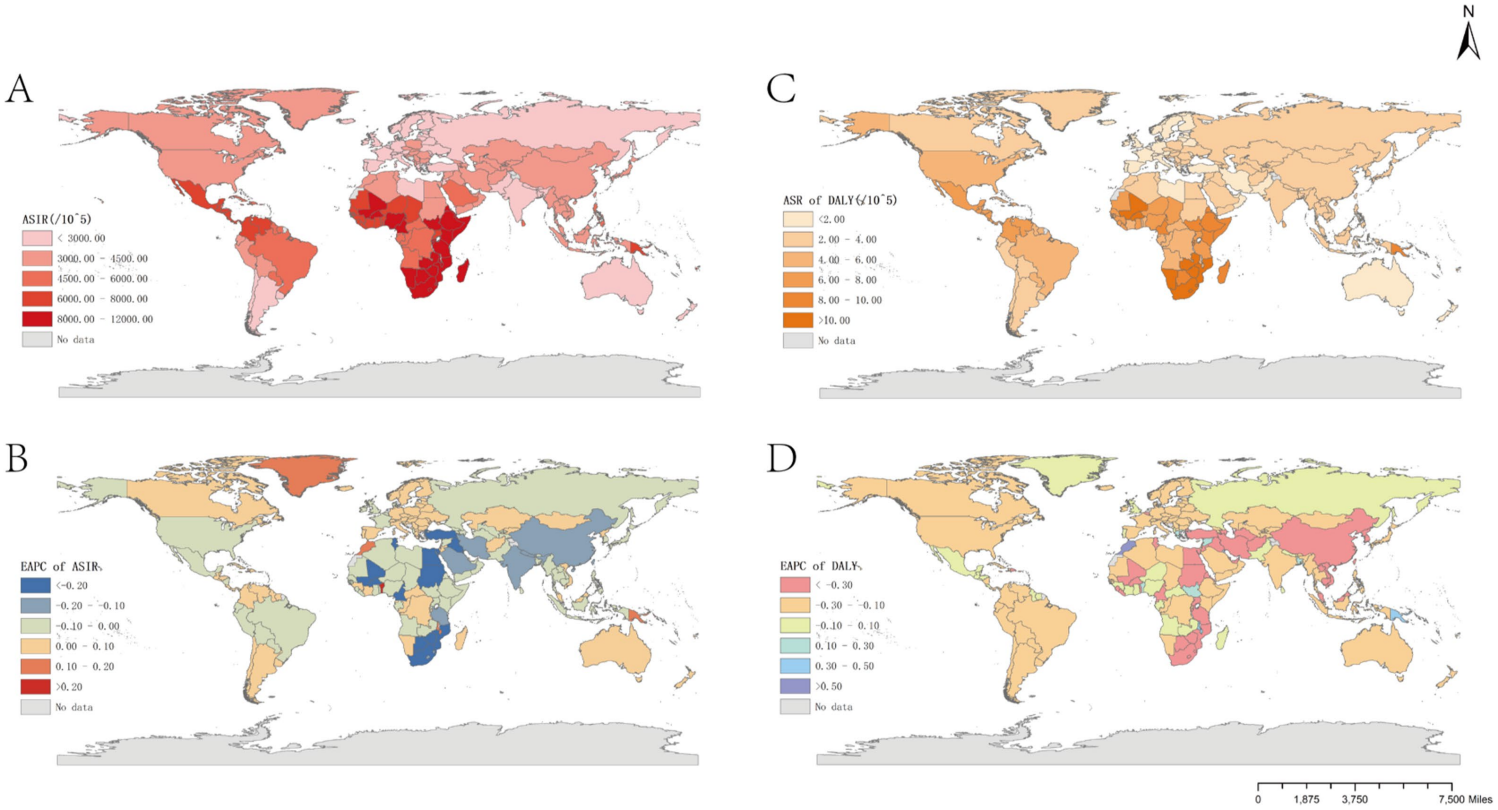
Global burden of trichomoniasis in 204 countries and territories. **(A)** ASIR in 2021. **(B)** EAPC for ASIR, 1990–2021. **(C)** ASR for DALY in 2021. **(D)** EAPC for ASR of trichomoniasis DALY, 1990–2021. ASIR, age-standardized incidence rate; EAPC, estimated annual percentage change.

The burden of trichomoniasis infection varies significantly between countries. The three countries with the highest ASIRs in 2021 are the United Republic of Tanzania, Zambia and Mozambique (Table 1 and Figure 3). China had the highest number of infections in 2021 (65,088,138) and the lowest was Tokelau (91). The highest ASIR is found in Eastern Sub-Saharan Africa, with the lowest being found in Western Europe (Table 1 and Figure 3). The ASIR by an average of 0.09 (95% CI: 0.06 to 0.13) per annum over the same period (from 3,950.01/100,000 in 1990 to 4,133.41/100,000 in 2021). The largest increase in ASIR was in Benin (EAPC = 0.27; 95% CI: 0.09 to 0.46), next is Morocco, and largest ASIR reduction was in South Africa (EAPC = −0.68). The highest ASIR and ASR for DALY 2021 are in Sub-Saharan Africa. The lowest ASIR and ASR for DALY in 2021 were in Western Europe.

### Joinpoint analysis results

As shown in Supplementary Figure S1, joinpoint regression analysis identified the age-specific trends in the burden of trichomoniasis in men from 1990 to 2021. The ASIR for overall trichomoniasis in men showed a fluctuating increase up to age 69, followed by a fluctuating decrease after wards. Similarly, as shown in Supplementary Figure S2, joinpoint regression analysis revealed that from 1990 to 2021, the ASIR for age-specific trichomoniasis in women increased with some volatility up to age 84, but declined precipitously after age 85. The fastest-growing ASIR occurred in the 25–29 age group from 2015 to 2019 (APC = 3.37).

ASIR trends in the 30–54 year-old female population align with the overall population’s ASIR trends.

### Incidence projections

**Figure 4:**
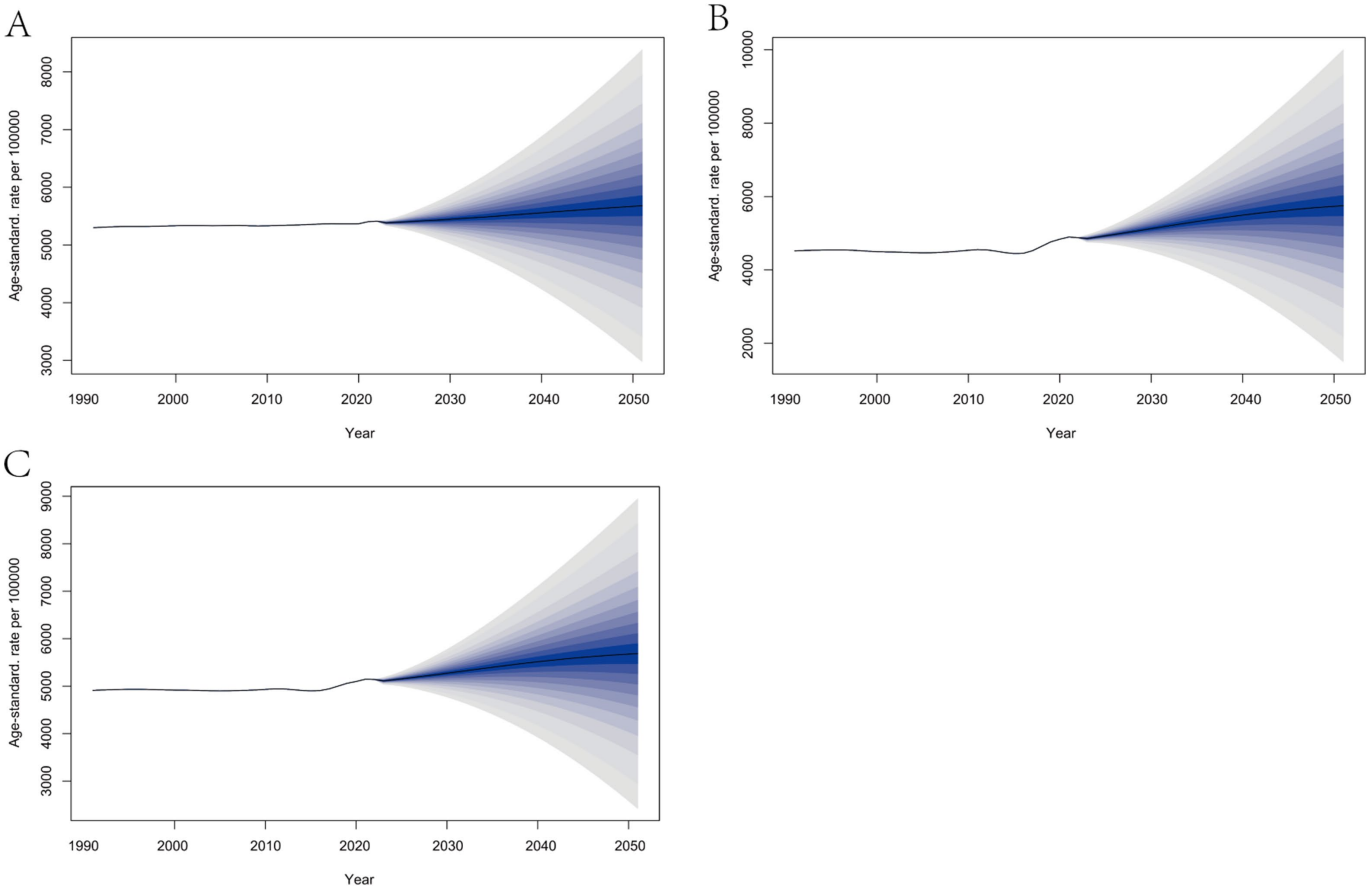
Projected incidence of trichomoniasis, 2022–2050. **(A)** Incidence in males. **(B)** Incidence in females. **(C)** Total incidence.

The projected incidence of trichomoniasis from 2022 to 2050 was calculated based on trichomoniasis burden data from 1990 to 2021. The incidence for 2022 to 2050 was projected by sex using Bayesian age-period-cohort analysis. The findings show that overall, overall, the incidence of trichomoniasis is expected to increase significantly in the future, with a notable gender difference (Figure 4). The projected number of cases by 2050 is 4,873.70×10^5^, with an ASIR projected to be 5,832.60 per 100,000 people.

The incidence rate for males levels off, while the incidence rate for females increases significantly. Although the ASIR for males remains much higher than for females, it is expected that the ASIR for females will catch up with that of males by 2043. The ASIR for males is projected to be 5,680.57 per 100,000, and for females, 5,749.47 per 100,000 in 2050.

## Discussion

This study provides a synthesis of global trichomoniasis burden estimates over the past 32 years. Between 1990 and 2021, both the incidence of trichomoniasis and its DALYs (Disability-Adjusted Life Years) increased. The primary factor contributing to this trend is likely the rapid growth of the world population, which has led to a larger population base. Trichomoniasis also exhibited elevated age-standardized incidence rates (ASIR) and age-standardized rates for DALY (ASR) over this period. A key reason for the increasing burden may be that most trichomoniasis cases are asymptomatic (20). Consequently, many individuals do not seek diagnosis or treatment, which facilitates the disease’s spread among sexual partners (21).

The transmission and prevalence of trichomoniasis exhibit significant differences between males and females. The ASIR indicates that it is higher in males than in females, while the ASR of DALY shows that it is higher in females than in males. This conclusion is consistent with the research findings of Leiwen Fu (15) and those of Poole et al. (22).

The differences in infection rates may be due to both behavioral and physiological factors. Men tend to change partners more frequently and have earlier sexual initiation compared to women (23). However, women are more severely affected by trichomoniasis because of their unique pathophysiology, which increases their risk of infertility, cervicitis, and cervical cancer following infection (4, 6, 7). Although men are often asymptomatic, their ability to unknowingly transmit the infection highlights the need for more accessible and responsive testing for men (24).

The burden of trichomoniasis also varies by age group. We observed the most significant change in ASIR among the <20 age group, likely due to the initially low ASIR in this group, making any increase more noticeable. A negative Estimated Annual Percentage Change (EAPC) and an increasing number of cases in the 85+ age group suggest a rise in the aging population. Furthermore, the negative EAPC and increasing cases in this age group suggest that the incidence of trichomoniasis is not under control, but rather that the ASIR is declining due to the growing proportion of older adult individuals in the global population (25). These trends imply that as the global population continues to grow, trichomoniasis infections will likely rise, underscoring the need for enhanced preventive and control strategies. In 2021, the 30–44 age group exhibited significantly higher ASRs for both incidence and DALY compared to other age groups. This may be due to an increase in sexual partners and unsafe sexual practices, which is consistent with findings from a British study that identified age as an independent risk factor for *Trichomonas vaginalis* infection (26).

Another major finding from the current study is that the ASIR and ASR for DALY associated with trichomoniasis decreased with higher levels of the Socio-Demographic Index (SDI). This supports the work of Zheng et al. (27). SDI is a reflection of a nation’s or region’s economic development, and the results suggest that trichomoniasis remains a persistent issue in low-income areas. Developing countries, particularly in sub-Saharan Africa, have higher DALY rates, indicating they bear the heaviest burden of trichomoniasis. Possible contributing factors include better early diagnosis and treatment in developed countries, and a lack of awareness and active treatment in developing regions (28). However, it is worth noting that EAPC for ASIRs is negative in low-SDI areas, while EAPC for ASIRs is positive in high-SDI areas. In economically disadvantaged regions, asymptomatic or mildly symptomatic infections may be underreported as resources are directed toward more urgent health concerns. In contrast, higher-income areas may experience better disease control measures, leading to lower case numbers, but resulting in a higher EAPC when ASIRs increase.

Regional differences in the ASIRs of trichomoniasis are also quite remarkable. The incidence rate is relatively high in African countries, followed by that in Oceania and Latin America. Cultural beliefs and lifestyle factors may contribute to these disparities (29). It has been demonstrated that condom use, as a reliable method of preventing STIs, significantly reduces the rates of trichomoniasis transmission (30). However, in regions with limited sexual health education, lower condom usage results in higher ASIRs. For instance, despite efforts to improve healthcare, Benin still experiences one of the highest EAPCs, likely due to inadequate public health infrastructure (31).

Joinpoint analysis revealed differences in trends across age groups and between sexes. In men, the highest ASIR occurred in the 35–39 age group in 2003, while women reached their peak ASIR in the same age group in 2019. This suggests that the 35–39 age group represents the highest risk for developing trichomoniasis. Since women aged 30–54 accounted for 79.96% of the total incidence, and the burden of trichomoniasis in this group closely mirrors the overall population trend, this age group plays a significant role in determining the total burden of disease. These findings align with the study by Asmah et al. (32), which found a stronger correlation between trichomoniasis and age in women compared to men, with the older adult being less affected.

Our predictions for the future show a steady trend in ASIR for men, but a sharp increase in ASIR for women. This may reflect greater vulnerability in women, compounded by lower education levels and poorer financial status.

Based on the Global Burden of Disease (GBD) 2021 study, this research outlines the ongoing global burden of trichomoniasis and provides projections through 2050. However, this study has limitations. The GBD database is heavily concentrated in economically developed countries, and some poorer or developing regions may lack sufficient health data. Furthermore, because trichomoniasis is not a notifiable disease and many cases are asymptomatic, its true prevalence may be underreported. This study highlights the increasing burden of trichomoniasis over the past 32 years and projects its continued rise through 2050, offering valuable insights to help authorities in different countries and regions develop effective prevention and control strategies.

## Conclusion

The global burden of trichomoniasis is rising. While men have higher ASIRs, women bear a greater DALY burden, highlighting the need for expanded testing options for men and more effective treatments for women, particularly those aged 30–54. The study revealed an inverse correlation between ASIR and SDI, indicating that trichomoniasis remains prevalent in low-income areas. Projections suggest a continued increase in global ASIR by 2050, especially among women. To mitigate this burden, strengthening sexual health education and STI surveillance in high-risk populations and regions is essential.

## Data Availability

Publicly available datasets were analyzed in this study. This data can be found here: https://ghdx.healthdata.org/gbd-2021.

## Footnotes

^1^ https://ghdx.healthdata.org/gbd-2021

^2^ https://surveillance.cancer.gov/help/joinpoint/tech-help/citation

## Supplementary material

SUPPLEMENTARY FIGURE S1 Joinpoint regression analyses for different male age subgroups. **(A)** <20 years; **(B)** 20–24 years; **(C)** 25–29 years; **(D)** 30–34 years; **(E)** 35–39 years; **(F)** 40–44 years; **(G)** 45–49 years; **(H)** 50–54 years; **(I)** 55–59 years; **(J)** 60–64 years; **(K)** 65–69 years; **(L)** 70–74 years; **(M)** 75–79 years; **(N)** 80–84 years; **(O)** 85+ years. APC, Annual Percent Change.

SUPPLEMENTARY FIGURE S2 Joinpoint regression analyses for different female age subgroups. **(A)** <20 years; **(B)** 20–24 years; **(C)** 25–29 years; **(D)** 30–34 years; **(E)** 35–39 years; **(F)** 40–44 years; **(G)** 45–49 years; **(H)** 50–54 years; **(I)** 55–59 years; **(J)** 60–64 years; **(K)** 65–69 years; **(L)** 70–74 years; **(M)** 75–79 years; **(N)** 80–84 years; **(O)** 85+ years. APC, Annual Percent Change.
