# Supplementary figures and images for: Global burden of trichomoniasis: current status, trends, and projections (1990–2021)
Source: Front Public Health. 2025 Feb 28;13:1530227. doi: 10.3389/fpubh.2025.1530227 (PMC11906697; doi:10.3389/fpubh.2025.1530227)

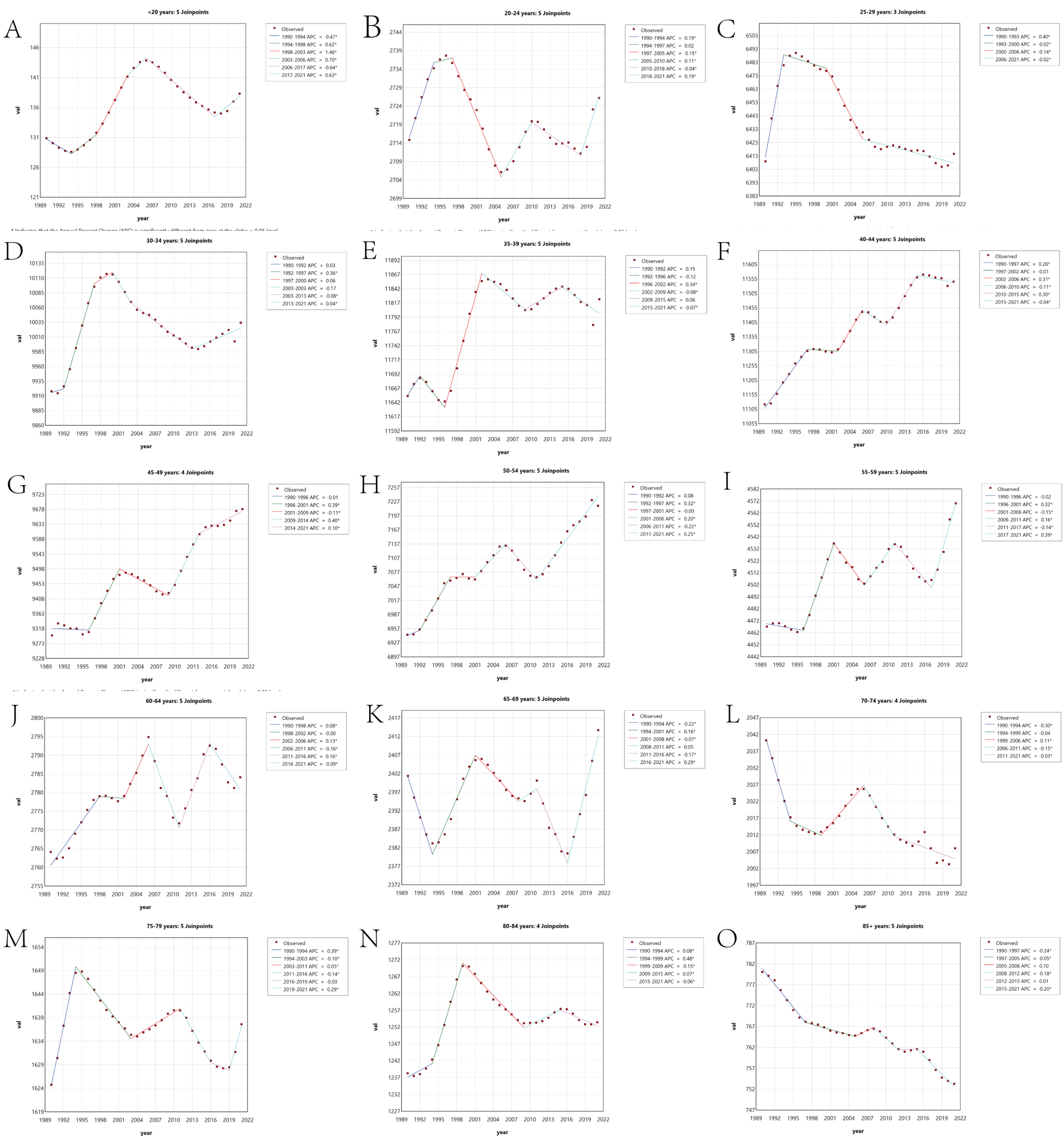

Supplement: SUPPLEMENTARY FIGURE S1 — Joinpoint regression analyses for different male age subgroups. (A) <20 years; (B) 20–24 years; (C) 25–29 years; (D) 30–34 years; (E) 35–39 years; (F) 40–44 years; (G) 45–49 years; (H) 50–54 years; (I) 55–59 years; (J) 60–64 years; (K) 65–69 years; (L) 70–74 years; (M) 75–79 years; (N) 80–84 years; (O) 85+ years. APC, Annual Percent Change. [file Data_Sheet_1.PDF]

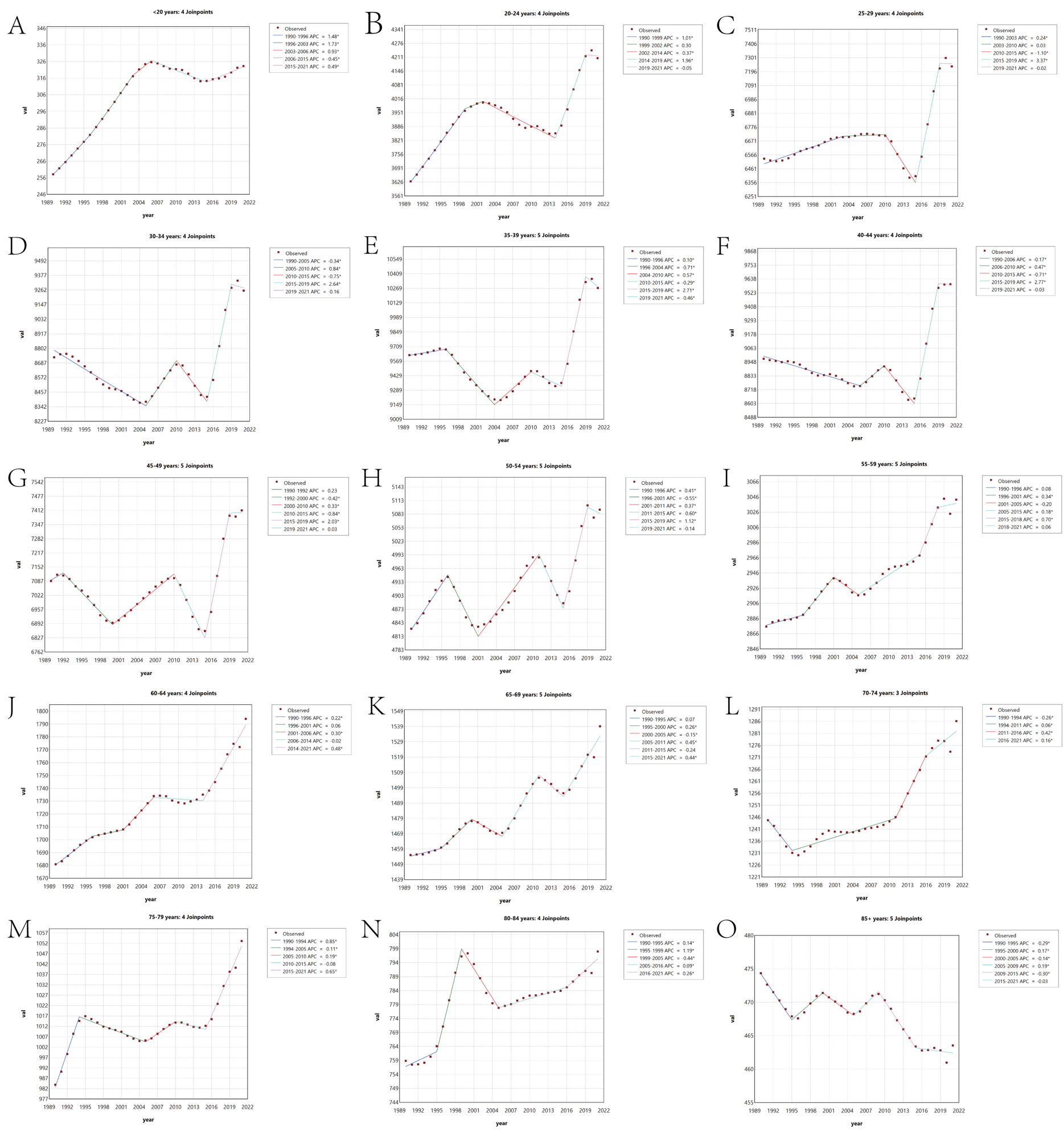

Supplement: SUPPLEMENTARY FIGURE S2 — Joinpoint regression analyses for different female age subgroups. (A) <20 years; (B) 20–24 years; (C) 25–29 years; (D) 30–34 years; (E) 35–39 years; (F) 40–44 years; (G) 45–49 years; (H) 50–54 years; (I) 55–59 years; (J) 60–64 years; (K) 65–69 years; (L) 70–74 years; (M) 75–79 years; (N) 80–84 years; (O) 85+ years. APC, Annual Percent Change. [file Data_Sheet_2.PDF]
