# Supplementary material for: Global burden of trichomoniasis: current status, trends, and projections (1990–2021)
Source: Front Public Health. 2025 Feb 28;13:1530227. doi: 10.3389/fpubh.2025.1530227 (PMC11906697; doi:10.3389/fpubh.2025.1530227)
Supplement: Supplementary file 3 [file Table_1.DOCX]

**Table S1:** **Trichomoniasis DALYs, rates and their temporal trends, 1990 and 2021**

| Characteristics | 1990 | | 2021 | | 1990 to 2021 |
| --- | --- | --- | --- | --- | --- |
|  | DALY ×10^3^  (95% UI) | ASR /100,000  No. (95% UI) | DALY ×10^3^  (95% UI) | ASR /100,000  No. (95% UI) | EAPC (95% CI) |
| Global | 158.91  (65.13 to 342.34) | 3.15 (1.29 to 6.76) | 274.36  (113.21 to 583.90) | 3.33 (1.37 to 7.12) | 0.03 (-0.03 to 0.09) |
| Sex |  |  |  |  |  |
| Female | 153.33  (62.72 to 330.58) | 6.1 (2.51 to 13.17) | 264.80  (109.09 to 565.05) | 6.45 (2.65 to 13.87) | 0.01 (-0.05 to 0.08) |
| Male | 5.58  (2.21 to 12.16) | 0.22 (0.09 to 0.48) | 9.55  (3.83 to 20.62) | 0.23 (0.09 to 0.5) | 0.08 (0.07 to 0.08) |
| Age |  |  |  |  |  |
| <20 years | 5.30  (1.76 to 12.03) | 0.23 (0.08 to 0.53) | 7.85  (2.59 to 18.05) | 0.3 (0.1 to 0.68) | 0.71 (0.52 to 0.9) |
| 20-24 years | 17.52  (6.05 to 39.79) | 3.56 (1.23 to 8.09) | 24.54  (8.50 to 55.69) | 4.11 (1.42 to 9.33) | 0.22 (0.09 to 0.35) |
| 25-29 years | 26.80  (9.16 to 59.36) | 6.05 (2.07 to 13.41) | 38.61  (13.13 to 86.49) | 6.56 (2.23 to 14.7) | 0.05 (-0.06 to 0.15) |
| 30-34 years | 26.65  (8.67 to 59.67) | 6.91 (2.25 to 15.48) | 43.70  (14.11 to 97.70) | 7.23 (2.33 to 16.16) | -0.04 (-0.14 to 0.05) |
| 35-39 years | 24.73  (8.38 to 58.14) | 7.02 (2.38 to 16.5) | 41.28  (14.07 to 97.18) | 7.36 (2.51 to 17.33) | -0.05 (-0.17 to 0.07) |
| 40-44 years | 18.96  (6.34 to 45.55) | 6.62 (2.21 to 15.9) | 34.54  (11.44 to 83.66) | 6.9 (2.29 to 16.72) | -0.05 (-0.14 to 0.05) |
| 45-49 years | 12.87  (4.10 to 30.78) | 5.54 (1.77 to 13.26) | 26.73  (8.45 to 62.76) | 5.65 (1.79 to 13.25) | -0.05 (-0.1 to 0.01) |
| 50-54 years | 9.15  (2.72 to 22.80) | 4.3 (1.28 to 10.73) | 19.85  (5.87 to 49.58) | 4.46 (1.32 to 11.14) | 0.04 (0 to 0.07) |
| 55-59 years | 6.33  (1.98 to 14.48) | 3.42 (1.07 to 7.82) | 14.15  (4.37 to 31.82) | 3.58 (1.11 to 8.04) | 0.11 (0.09 to 0.14) |
| 60-64 years | 4.19  (1.53 to 9.28) | 2.61 (0.95 to 5.78) | 8.65  (3.12 to 19.30) | 2.7 (0.98 to 6.03) | 0.14 (0.1 to 0.17) |
| 65-69 years | 2.87  (1.09 to 6.18) | 2.32 (0.88 to 5) | 6.37  (2.44 to 13.60) | 2.31 (0.88 to 4.93) | 0.04 (0.01 to 0.08) |
| 70-74 years | 1.75  (0.67 to 3.74) | 2.07 (0.79 to 4.42) | 4.05  (1.56 to 8.72) | 1.97 (0.76 to 4.24) | -0.1 (-0.15 to -0.06) |
| 75-79 years | 1.07  (0.43 to 2.21) | 1.74 (0.7 to 3.59) | 2.20  (0.88 to 4.48) | 1.67 (0.66 to 3.4) | -0.19 (-0.24 to -0.15) |
| 80-84 years | 0.51  (0.21 to 1.04) | 1.44 (0.59 to 2.95) | 1.20  (0.49 to 2.43) | 1.37 (0.56 to 2.77) | -0.2 (-0.23 to -0.17) |
| 85+ years | 0.21  (0.08 to 0.44) | 1.03 (0.41 to 2.17) | 0.645  (0.25 to 1.35) | 0.94 (0.37 to 1.95) | -0.24 (-0.29 to -0.19) |
| SDI |  |  |  |  |  |
| Low SDI | 22.84  (9.36 to 46.71) | 5.77 (2.43 to 11.97) | 52.92  (21.71 to 110.14) | 5.53 (2.28 to 11.63) | -0.24 (-0.29 to -0.19) |
| Low-middle SDI | 30.86  (12.62 to 64.58) | 3.18 (1.3 to 6.8) | 64.65  (26.59 to 139.38) | 3.36 (1.38 to 7.18) | -0.03 (-0.11 to 0.04) |
| Middle SDI | 54.19  (22.10 to 117.24) | 3.41 (1.41 to 7.29) | 91.50  (37.69 to 192.29) | 3.38 (1.39 to 7.16) | -0.21 (-0.28 to -0.13) |
| High-middle SDI | 25.24  (10.38 to 55.03) | 2.28 (0.95 to 4.89) | 33.70  (14.03 to 69.63) | 2.18 (0.9 to 4.63) | -0.35 (-0.45 to -0.25) |
| High SDI | 25.61  (10.55 to 53.73) | 2.61 (1.07 to 5.48) | 31.32  (13.02 to 64.50) | 2.52 (1.03 to 5.31) | -0.15 (-0.17 to -0.12) |
| Region |  |  |  |  |  |
| Andean Latin America | 1.11  (0.45 to 2.31) | 3.52 (1.42 to 7.42) | 2.32  (0.96 to 4.80) | 3.39 (1.4 to 6.98) | -0.22 (-0.26 to -0.19) |
| Australasia | 0.40  (0.17 to 0.83) | 1.82 (0.76 to 3.75) | 0.62  (0.26 to 1.26) | 1.78 (0.74 to 3.73) | -0.11 (-0.14 to -0.08) |
| Caribbean | 1.57  (0.63 to 3.31) | 4.81 (1.97 to 10.12) | 2.37  (0.99 to 4.81) | 4.71 (1.99 to 9.61) | -0.14 (-0.16 to -0.11) |
| Central Asia | 2.49  (0.99 to 5.09) | 4.01 (1.64 to 8.25) | 3.81  (1.58 to 7.84) | 3.79 (1.57 to 7.77) | -0.2 (-0.23 to -0.17) |
| Central Europe | 3.46  (1.45 to 7.23) | 2.57 (1.07 to 5.4) | 3.28  (1.40 to 6.66) | 2.44 (1.02 to 5.04) | -0.19 (-0.23 to -0.15) |
| Central Latin America | 9.77  (3.95 to 20.98) | 7.09 (2.93 to 14.95) | 18.92  (7.75 to 40.03) | 7.04 (2.88 to 14.91) | -0.08 (-0.12 to -0.05) |
| Central Sub-Saharan Africa | 1.90  (0.77 to 3.86) | 4.55 (1.87 to 9.35) | 4.82  (2.00 to 9.94) | 4.31 (1.8 to 8.84) | -0.19 (-0.21 to -0.17) |
| East Asia | 31.35  (12.68 to 68.67) | 2.56 (1.05 to 5.54) | 42.81  (17.73 to 89.45) | 2.44 (1.01 to 5.25) | -0.37 (-0.54 to -0.2) |
| Eastern Europe | 5.23  (2.15 to 11.04) | 2.08 (0.86 to 4.39) | 5.04  (2.10 to 10.48) | 2.04 (0.84 to 4.26) | -0.08 (-0.12 to -0.05) |
| Eastern Sub-Saharan Africa | 13.50  (5.51 to 27.52) | 9.18 (3.85 to 18.94) | 31.64  (12.76 to 65.47) | 8.65 (3.5 to 18.11) | -0.3 (-0.37 to -0.24) |
| High-income Asia Pacific | 4.49  (1.86 to 9.37) | 2.3 (0.95 to 4.87) | 4.67  (1.92 to 9.62) | 2.16 (0.88 to 4.54) | -0.25 (-0.29 to -0.21) |
| High-income North America | 13.54  (5.47 to 29.12) | 4.32 (1.74 to 9.2) | 16.41  (6.69 to 34.37) | 4.09 (1.66 to 8.74) | -0.15 (-0.2 to -0.1) |
| North Africa and Middle East | 6.7  (2.74 to 14.04) | 2.45 (1.02 to 5.08) | 14.64  (6.04 to 31.14) | 2.26 (0.92 to 4.73) | -0.75 (-0.95 to -0.55) |
| Oceania | 0.49  (0.20 to 1.00) | 8.71 (3.59 to 18.02) | 1.1  (0.49 to 2.47) | 8.98 (3.77 to 18.76) | 0.41 (0.22 to 0.59) |
| South Asia | 19.04  (7.73 to 40.68) | 2.04 (0.84 to 4.41) | 39.05  (15.96 to 85.56) | 2.04 (0.84 to 4.41) | -0.17 (-0.28 to -0.06) |
| Southeast Asia | 13.46  (5.49 to 28.74) | 3.31 (1.38 to 6.96) | 23.26  (9.79 to 48.43) | 3.05 (1.28 to 6.35) | -0.28 (-0.29 to -0.26) |
| Southern Latin America | 1.06  (0.44 to 2.24) | 2.2 (0.91 to 4.67) | 1.57  (0.66 to 3.24) | 2.09 (0.88 to 4.35) | -0.16 (-0.2 to -0.12) |
| Southern Sub-Saharan Africa | 6.09  (2.49 to 12.78) | 13.37 (5.49 to 28.8) | 9.55  (3.91 to 20.53) | 11.4 (4.66 to 24.68) | -1.09 (-1.37 to -0.79) |
| Tropical Latin America | 7.46  (3.01 to 16.21) | 5.44 (2.21 to 11.67) | 13.70  (5.63 to 28.97) | 5.34 (2.17 to 11.29) | -0.14 (-0.22 to -0.06) |
| Western Europe | 4.93  (2.07 to 10.14) | 1.13 (0.48 to 2.37) | 5.42  (2.30 to 10.86) | 1.09 (0.46 to 2.23) | -0.11 (-0.14 to -0.09) |
| Western Sub-Saharan Africa | 10.81  (4.43 to 22.13) | 7.14 (2.96 to 14.79) | 29.27  (12.02 to 61.15) | 7.41 (3.06 to 15.63) | -0.21 (-0.31 to -0.11) |

**DALY Disability-adjusted life year, EAPC estimated annual percentage change, 95% UI 95% uncertainty interval, 95% CI 95% confidence interval, SDI Socio-demographic Index**
